# Prevalence of Human T-Lymphotropic Viruses 1 and 2 in Individuals Infected with Hepatitis C Virus in Belém do Pará, Brazil

**DOI:** 10.3390/tropicalmed11040095

**Published:** 2026-04-02

**Authors:** Renata Santos de Sousa, Lorena de Carvalho Corrêa, Fabiola Santos da Silva Matos, Samia Meneses dos Santos, Marcos Daniel Mendes Padilha, Carolina Cabral Angelim, Álesson Adam Fonseca Andrade, Amanda Roberta Vieira Sacramento, Aline Cecy Rocha de Lima, João Lukas Nunes Almeida, Mauro Sérgio Moura de Araújo, Vitória Sahena Martins Souza Barbosa, Jacqueline Cortinhas Monteiro, Greice de Lemos Cardoso Costa, Andréa Nazaré Monteiro Rangel da Silva, Simone Regina Souza da Silva Conde, Luiz Fernando Almeida Machado, Izaura Maria Vieira Cayres Vallinoto, Antonio Carlos Rosário Vallinoto, Rosimar Neris Martins Feitosa

**Affiliations:** 1 Laboratório de Virologia, Instituto de Ciências Biológicas, Universidade Federal do Pará, Belém 66075-110, Brazil; renatadeeesousa@gmail.com (R.S.d.S.); lorena.correa@icb.ufpa.br (L.d.C.C.); fabiola.matos@icb.ufpa.br (F.S.d.S.M.); samiameneses31@gmail.com (S.M.d.S.); marcos.padilha@icb.ufpa.br (M.D.M.P.); carolinacabralangelim@gmail.com (C.C.A.); alinececy@gmail.com (A.C.R.d.L.); jacqueline@ufpa.br (J.C.M.); andrearangel@ufpa.br (A.N.M.R.d.S.); lfam@ufpa.br (L.F.A.M.); ivallinoto@ufpa.br (I.M.V.C.V.); 2 Programa de Pós-Graduação em Biologia de Agentes Infecciosos e Parasitários, Instituto de Ciências Biológicas, Universidade Federal do Pará, Belém 66075-110, Brazil; prof.alessonandrade.bio@gmail.com (Á.A.F.A.); greice@ufpa.br (G.d.L.C.C.); 3 Laboratório de Genética Humana e Médica, Instituto de Ciências Biológicas, Universidade Federal do Pará, Belém 66075-110, Brazil; roberta.amanda@hotmail.com; 4 Hospital Universitário João de Barros Barreto, Belém 66073-000, Brazil; joao.almeida@icb.ufpa.br (J.L.N.A.); araujomsm@hotmail.com (M.S.M.d.A.); sconde@ufpa.br (S.R.S.d.S.C.); 5 Centro de Atenção à Saúde nas Doenças Infecciosas Adquiridas (CASADIA), Belém 66113-190, Brazil; sahenabarbosa19@gmail.com; 6 Faculdade de Medicina, Instituto de Ciências da Saúde, Universidade Federal do Pará, Belém 66075-110, Brazil

**Keywords:** HTLV-1/2, HCV, coinfection, epidemiology

## Abstract

Coinfection between hepatitis C virus (HCV) and human T-lymphotropic virus 1/2 (HTLV-1/2) remains poorly investigated in the Northern Region of Brazil despite its clinically important condition. The objective of this study was to determine the prevalence and describe the epidemiological and behavioral risk factors for HCV/HTLV-1/2 coinfection in Belém, Pará. This observational, descriptive, and cross-sectional study analyzed 192 samples from patients previously diagnosed with HCV: 127 participants recruited between May 2023 and June 2025 and 65 samples previously stored in the Virology Laboratory of UFPA. Data were collected through a structured survey. Serological screening for HTLV-1/2 was performed by enzyme-linked immunosorbent assay (ELISA) and confirmed by INNO-LIA and molecular biology (qPCR). HCV/HTLV-1/2 coinfection was observed in 4 individuals (2.1%), of whom 1.6% had HCV/HTLV-1 coinfection and 0.5% HCV/HTLV-2. There was no statistically significant association when comparing the sociodemographic, clinical characteristics, or risk factors of HCV monoinfected and HCV/HTLV-1/2 coinfected individuals. Although the results show a low prevalence of HTLV-1/2 and HCV coinfection in Belém do Pará, they still reinforce the importance of including HTLV in testing protocols for patients with hepatitis C in the North region of Brazil.

## 1. Introduction

Infections caused by the hepatitis C virus (HCV) and by Human T-lymphotropic viruses 1 and 2 (HTLV-1/2) remain unclear and poorly investigated in global landscape. Studies report controversial clinical outcomes during HTLV/HCV coinfection. In studies related to HCV/HTLV coinfection, Campos et al., 2020 [1], showed that individuals coinfected with HCV/HTLV-1 had higher viral loads and lower rates of HCV clearance, whereas individuals coinfected with HCV/HTLV-2 had lower viral loads and higher rates of HCV clearance compared to patients monoinfected with HCV. In another study, a reduction in liver damage was observed in individuals co-infected with HCV/HTLV-1, suggesting that this co-infection reduces acute immune-mediated damage in hepatocytes infected with HCV, as demonstrated by the presence of higher levels of Th1-type cytokines and CD4+ T lymphocytes, lower levels of liver fibrosis, and liver enzymes [2].

HCV is transmitted predominantly through parenteral exposure, including the use of injectable drugs [3], as well as through sexual (mainly from male to female) [4] and vertical transmission [5]. Chronic HCV infection may progress to hepatic cirrhosis and hepatocellular carcinoma [6,7,8]. Surveillance data identify hepatitis C as one of the main viral infections monitored in the country (476,528 cases between 2000 and 2024), with the highest concentration of cases occurring in the Southeast Region (50%). Although the Northern Region, including the state of Pará, presents one of the lowest prevalence rates compared with other regions (5.5%), records still indicate ongoing transmission in this territory [9].

Human T-lymphotropic Viruses 1 and 2 (HTLV-1/2) were first described more than 40 years ago [10,11]. Transmission occurs through sexual contact, breastfeeding from an infected mother, sharing of sharp instruments, and blood transfusion [12,13,14,15,16,17]. While many individuals remain asymptomatic, HTLV-1 is associated with potentially severe conditions including HTLV-1-associated tropical spastic paraparesis/myelopathy (HAM/TSP) [18,19], adult T-cell leukemia/lymphoma (ATLL) [20,21], uveitis [22], dermatitis [23], conjunctivitis, sicca syndrome, interstitial keratitis, Sjögren’s syndrome, Hashimoto’s thyroiditis, Graves’ disease, pulmonary disease, inflammatory myositis, and arthritis [24].

HTLV-1/2 has global distribution, impacting an estimated 10 million individuals. These viruses are considered endemic in regions such as Japan, Iran, sub-Saharan Africa, the Caribbean, the southeastern United States, and South America [25], including Brazil, within a distribution linked to historical and migratory factors [26]. Although not subject to systematic national surveillance, regional studies demonstrate the presence of these viruses in various populations, ranging from 0.19% to 29% in Northern region [27,28,29]. HTLV-1/2 infection has been identified in multiple vulnerable groups in the Amazon region, including Indigenous communities [30,31,32], Indigenous refugees [33], residents of the metropolitan area of Belém, Pará [29,34,35], Japanese immigrants [36], quilombola communities [37,38] and riverine and rural populations [39].

**Figure 1:**
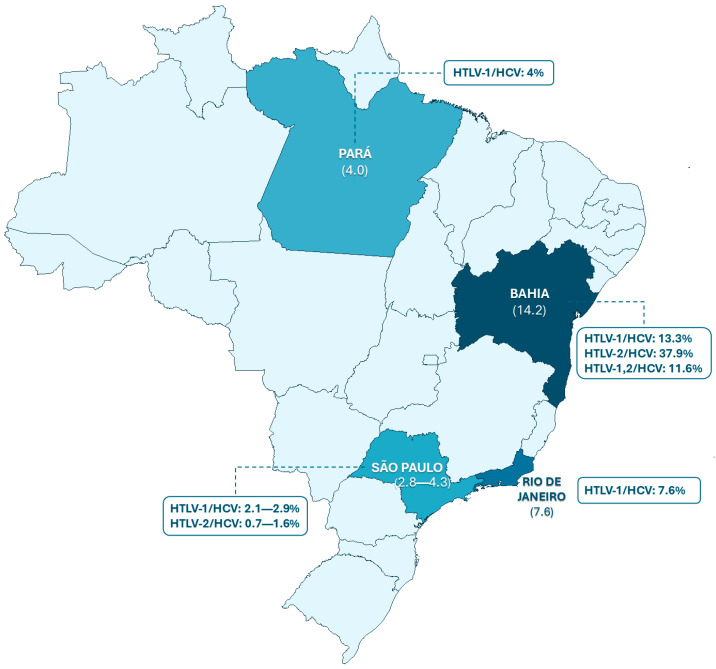
Prevalence of HTLV-1/2 and HCV coinfection across Brazilian states. Source: São Paulo [1,40,41], Bahia [42], Pará [43] and Rio de Janeiro [2].

Coinfection with HCV and HTLV-1/2 has already been documented in certain settings in Brazil, particularly in studies conducted in São Paulo (prevalence ranging from 2.8% to 4.3%) [1,40,41], Bahia (14.2%) [42], Rio de Janeiro (7.6%) [2], and Pará (4.0%) [43] as shown in Figure 1. Despite these results, reports in the Northern Region, including the state of Pará, remain limited, highlighting a significant gap in understanding the combined epidemiology of these viruses. The absence of updated information in Belém compromises the assessment of the magnitude of the issue and hinders the planning of surveillance and prevention strategies aimed at vulnerable populations.

Given the significance of this research topic, the present study aimed to determine the prevalence of HCV/HTLV-1/2 coinfection and to describe associated risk behaviors among individuals receiving healthcare in Belém, Pará.

## 2. Materials and Methods

### 2.1. Study Area and Data Collection

This observational, descriptive, and cross-sectional study analyzed 192 samples from patients with a previously confirmed diagnosis of HCV infection, sourced from three healthcare facilities located in the city of Belém, Pará, Brazil. The state of Pará comprises a total area of 1,248,000 km^2^, whereas the capital, Belém, a predominantly urban area, encompasses 1059.4 km^2^ with a population of approximately 2.4 million people [44].

Whole blood samples were collected from 127 individuals of both sexes, aged 18 years and above, between May 2023 and June 2025. Participation in the study was voluntary; participants were duly informed about the study description and invited to participate. After learning about the study and consenting the use of their samples, they signed an informed consent form and completed an epidemiological survey containing sociodemographic and behavioral questions about risk for HTLV-1/2 infection. Sixty-five (65) blood samples previously stored in the Virology Laboratory of UFPA were also analyzed. Clinical (cirrhosis and HCV viral load) and laboratory (ALT, AST, and GGT) information was obtained from physical medical records or electronically accessed databases, as authorized by the institutions involved.

### 2.2. Ethics Statement

Ethical clearance for demographic data and whole blood sample collection was granted from the institutional review boards (IRB) from each healthcare facility including Santa Casa de Misericórdia Foundation (CAE: 31223214.2.3001.5171), João de Barros Barreto University Hospital and Centro de Atenção à Saúde nas Doenças Infecciosas Adquiridas—CASADIA (CAE: 65072922.6.0000.0018). Formal written consent was obtained from all participants, and all procedures were conducted in accordance with the principles outlined in the Declaration of Helsinki.

### 2.3. Serological Screening

Following national technical recommendations [45], plasma detection of total anti-HTLV-1/2 antibodies (anti-gp46 and anti-gp21) was performed using ELISA screening (MUREX HTLV I + II, Diasorin, Dietzenbach, Germany), in accordance with the manufacturer’s protocol. Samples that showed an absorbance lower than the cut-off value were considered non-reactive for HTLV. Samples with positive (absorbance values greater than or equal to the cut-off value) results were subjected to confirmatory testing using the Immunoblot methodology (INNO-LIA^®^ HTLV I/II Score, Fujirebio, Japan) for the detection of recombinant proteins of HTLV-1 (rgp46-I), HTLV-2 (rgp46-II), and gp21, an envelope epitope protein common to both viral types. In INNO-LIA a sample is considered “negative” when there are no reactive lines or only one reactive line, except for gp21. Samples are considered “positive” when there are two reactive lines in addition to that of gp21, or three or more reactive lines. The result is considered “indeterminate” when one reactive line (p19, p24, gp46, or gp21) or two reactive lines without reactivity for the gp21 appear. All samples with reactive (ELISA or Immunoblot) or indeterminate (Immunoblot) profiles were submitted to molecular analysis to confirm infection and determine HTLV typing. 

HCV infection was investigated using lateral-flow chromatography for the detection of anti-HCV antibodies. Reactive samples were subjected to viral RNA quantification to confirm active infection, in accordance with national laboratory diagnostic guidelines [46]. 

### 2.4. DNA Extraction

For HTLV proviral DNA testing, 200 µL of whole blood were used, which was subjected to DNA extraction using the QiaAmp DNA mini kit (Qiagen, Hilden, Germany) according to the manufacturer’s instructions. Prior to target amplification, samples were quantified using the Qubit 2.0 fluorimeter (Invitrogen, Carlsbad, CA, USA). Specimens considered valid for PCR should contain at least 20 ng of DNA. 

### 2.5. Confirmatory Diagnosis by Real-Time PCR (qPCR)

For molecular confirmation of infection, qPCR was performed using the TaqMan system (Applied Biosystems, Foster City, CA, USA) from three target sequences: the *albumin* gene, as an endogenous control, and the non-homologous regions of the *pol* gene of HTLV-1 and HTLV-2 [47]. The primer sequences in the 5′-3′ direction were GAAC-GCTCTAATGGCATTCTTAAAACC for HTLV-1F, GTGGTTGATTGTCCA-TAGGGCTAT for HTLV-1R, CAACCCCACCAGCTCAGG for HTLV-2F, GGGAAGGTTAGGACAGTCTAGTAGATA for HTLV-2R, GCTCAACTCCCTATTGCTATCACA for Albumin F and GGGCATGACAGGTTTT-GCAATATTA for Albumin R. The sequences of the probes used in the qPCR reaction in the 5′-3′ direction were FAM-ACAAACCCGACCTACCC-NFQ for HTLV-1, FAM-TCGAGAGAACCAATGGTATAAT-NFQ for HTLV-2 and FAM-TTGTGGGCTGTAATCAT-NFQ for albumin.

### 2.6. Statistical Analysis

Excel software (Microsoft Office Excel^®^ 2021, version 2603) was used to store epidemiological data. Statistical analysis was performed with BioEstat 5.3 and R version 4.2.2 (R Core, 2023). Descriptive statistics were organized in tables containing the relative and absolute frequencies of the investigated variables. Inferential analysis compared risk factors in two groups: individuals with HCV monoinfection and those with HCV/HTLV-1/2 coinfection. Proportions with respective 95% confidence intervals were estimated. The nonparametric test used was Fisher’s Exact Test, adopting a significant level of 5% (*p* < 0.05). Unreported variables were not included in the statistical analysis.

## 3. Results

A total of 192 individuals infected with HCV participated in the study. The investigation demonstrated a prevalence of HCV/HTLV-1/2 coinfection of 2.1% (4/192; 95% CI 0.6–5.3%), with 1.6% (3/192; 95% CI 0.3–4.5%) for HTLV-1 and 0.5% (1/192; 95% CI 0.0–2.9%) for HTLV-2.

Table 1 presents the sociodemographic characteristics of the participants. Among those coinfected, three (75%) were treated at the FSCMPA health facility. Among the four patients coinfected with HCV/HTLV-1/2, there was a balanced distribution between the sexes (two men and two women). Ages ranged from 35 to 72 years. Three coinfected individuals resided in Belém, two of whom were married, and one was single. Most data on ethnicity, education, and family income were not recorded. Only one reported being mixed-race, having incomplete primary education, and a family income of one minimum wage.

Table 2 describes the sociodemographic characteristics and results of laboratorial screening and confirmatory tests for HTLV-1/2 of individuals coinfected with HCV and HTLV-1/2. To identify HTLV-1/2 infection, ELISA screening was performed, followed by confirmatory tests (INNO-LIA and qPCR) on reactive samples. However, one sample was negative in INNO-LIA but presented detectable DNA by qPCR for HTLV-2. In another case, there was reactivity in INNO-LIA for HTLV-1, without detection of the *pol* gene by qPCR.

Regarding the patient characteristics, patient 1 was a 35-year-old male, born and residing in Belém, married, an alcoholic, had surgery before 1993, used injectable and inhalant drugs, and had tattoos. He had comorbidities, including pulmonary tuberculosis and chronic hepatitis C without cirrhosis. Patient 2 was a 66-year-old male with comorbidities including diabetes mellitus, benign prostatic hypertension, and chronic hepatitis C without cirrhosis. Patient 3 was a 50-year-old female, born and residing in Belém, married, had a blood transfusion before 1993, and underwent surgery after 1993. She had comorbidities, including hepatic steatosis and systemic arterial hypertension (SAH), as well as chronic hepatitis C without cirrhosis. Patient 4 was born and lives in Belém, was 72 years old, female, mixed race, single, had an incomplete high school education, had a blood transfusion before 1993, was breastfed, and has a child who was breastfed for six or more months.

Of the four coinfected individuals, only one presented alteration in ALT and AST, two presented elevated GGT levels, and all were reactive for anti-HCV and had a detectable HCV viral load.

Risk factors for infections are presented in Table 3. Among the co-infected individuals, two patients (50%) reported receiving blood transfusion prior 1993. Illicit drug use was reported by one participant. All had detectable viral load for hepatitis, and three had reactive anti-HCV for more than six months, characterizing chronic hepatitis.

A comparative analysis was performed between the mono-infected and co-infected groups regarding risk factors. It was decided to emphasize the description of the co-infected group, in line with the objective of the present study and considering the relevance of the topic. In addition, none of the variables analyzed showed a statistically significant association with co-infection. 

## 4. Discussion

The 2.1% prevalence of HCV/HTLV-1/2 coinfection (1.6% HCV/HTLV-1; 0.5% HCV/HTLV-2) identified in Belém, Pará, was lower than that reported in São Paulo, where studies have described a 5.3% prevalence of HCV/HTLV-1/2 coinfection (3.2% HCV/HTLV-1; 2.1% HCV/HTLV-2) [48] and, from another investigation, where a 4.0% coinfection prevalence was identified (2.4% HCV/HTLV-1; 1.6% HCV/HTLV-2) [41]. These differences may be associated with higher population density, more intense viral circulation, and the diversity of transmission routes observed in large urban centers, which can increase exposure to multiple risk factors.

In Bahia, a high prevalence of HCV/HTLV-2 coinfection (37.9%) has been reported [42], a pattern distinct from that observed in the present study, in which coinfection with HTLV-1 predominated. Bahia exhibits one of the highest HTLV-1/2 prevalence rates in Brazil [49]. It is believed that HTLV-1 entered the eastern region of the country through the transatlantic trafficking of enslaved individuals, whereas HTLV-2 is widely distributed among Indigenous peoples, although both viral types have African origins [26].

In the present study, coinfection was more frequently associated with HTLV-1, a pattern consistent with other studies conducted in the region that assessed HTLV-1 monoinfection [29], although more balanced proportions have also been documented [35,39]. This finding suggests that historical and sociodemographic factors may favor the circulation of HTLV-1 in the local population, influencing the dynamics of coinfection as well. Such regional differences in HTLV distribution support the hypothesis that its heterogeneity results from historical migration processes, population admixture, and variation in exposure to risk factors across regions.

In this study, one patient presented a negative INNO-LIA result for HTLV-1/2, whereas qPCR detected HTLV-2 proviral DNA. Studies indicate that confirmatory tests frequently yield indeterminate results for HTLV-1/2 infection, possibly due to low or absent expression of viral antigens that affect antibody-mediated immune responses [50,51,52,53]. Another patient showed a positive INNO-LIA result for HTLV-1, but qPCR did not detect proviral DNA. Confirmation of HTLV-1/2 infection may be hindered by factors such as mutations, incomplete viral particles, low quality of DNA extracted, and low proviral load [53], underscoring the importance of employing multiple diagnostic techniques [45]. Although, in the present investigation, the combined use of different laboratory methodologies minimized the risk of underestimating coinfection prevalence, it is important to note that studies relying on a single technique may miss cases, thereby affecting infection rate estimates and epidemiological interpretation.

Brazilian studies indicate that HTLV-1 may increase, whereas HTLV-2 may reduce, HCV viral load [1]. Additionally, patients coinfected with HTLV-2 exhibit higher clearance rates [41]. In the present study, this same pattern was observed, but one of the patients co-infected with HTLV-1 showed a reduced HCV viral load. Chronic hepatitis coinfection may reflect alterations in immune responses that favor viral persistence. However, the direction and impact of this interaction remain unclear, requiring additional studies to elucidate the immunological effects of coinfection.

Screening for HTLV became mandatory in Brazilian blood banks in 1993, and in 2024 the test was incorporated into prenatal care under the Unified Health System (SUS). Despite these advances, the virus remains neglected and lacks public policies aimed at prevention and control. The present study reinforces the importance of including HTLV-1/2 screening among individuals with HCV and represents one of the first reports of coinfection in the Northern Region of Brazil, where literature remains scarce. Only one record—a conference abstract presenting preliminary data on HCV/HTLV-1 coinfection in Belém, Pará—was identified [43]. Despite extensive research on HTLV in other populations, the current study holds social relevance by proposing coinfection screening and scientific relevance by generating novel epidemiological data, thereby helping to fill an important regional gap.

Sociodemographic and behavioral risk analyses in this study were limited by the absence of records for part of the samples, due to inconsistencies between stored data and information recently collected through epidemiological inquiry.

## 5. Conclusions

This study identified a low prevalence of HCV/HTLV-1/2 coinfection in the Amazon region, providing novel data to the local epidemiological context. Despite the absence of significant differences between monoinfected and coinfected participants regarding sociodemographic characteristics, classical risk factors, or clinical and virological parameters, both groups exhibited a predominantly male profile, advanced age, low educational attainment, and low income. These findings underscore a pattern of vulnerability to infectious diseases within this population, aligning with regional epidemiological reports. Furthermore, these results highlight the necessity for active surveillance, integrated serological screening, and the development of targeted public health policies to address these and other coinfections in the region. 

## Figures and Tables

**Table 1:** Sociodemographic characterization of individuals monoinfected with HCV and coinfected with HCV/HTLV-1/2 in Belém do Pará.

Variables	Total *n* = 192 (%)	HCV *n* = 188 (%)	HCV/HTLV-1/2 *n* = 4 (%)	*p*
Sex				
Male	106 (55.2)	104 (55.3)	2 (50.0)	1.000
Female	86 (44.8)	84 (44.7)	2 (50.0)	
Age Group				
20–29	10 (5.2)	10 (5.3)	0	0.5249
30–39	18 (9.4)	17 (9.0)	1 (25.0)	
40–49	43 (22.4)	43 (22.9)	0	
50–59	45 (23.4)	44 (23.4)	1 (25.0)	
≥60	76 (39.6)	74 (39.4)	2 (50.0)	
Skin color **				
White	5 (2.6)	5 (2.7)	0	- *
Black	21 (10.9)	21 (11.2)	0	
Brown	99 (51.6)	98 (52.1)	1 (25.0)	
Not informed	67 (34.9)	64 (34.0)	3 (75.0)	
Marital status				
Married	82 (42.7)	80 (42.6)	2 (50.0)	1.000
Single	69 (35.9)	68 (36.2)	1 (25.0)	
Separated/Divorced	19 (9.9)	19 (10.1)	0	
Widower	10 (5.2)	10 (5.3)	0	
Not informed	12 (6.3)	11 (5.9)	1 (25.0)	
Schooling level				
Elementary school	74 (38.5)	73 (38.9)	1 (25.0)	- *
High school	42 (21.9)	42 (22.3)	0	
Higher education	10 (5.2)	10 (5.3)	0	
Not informed	66 (34.4)	63 (33.5)	3 (75.0)	
Family income (minimum wage)				
<1	32 (16.7)	32 (17.0)	0	- *
1–2	72 (37.5)	71 (37.8)	1 (25.0)	
≥3	23 (12.0)	23 (12.2)	0	
Not informed	65 (33.8)	62 (33.0)	3 (75.0)	

* Statistical analysis was not performed because the expected frequencies were low. ** In Brazil, the term “skin color” is used by the Brazilian Institute of Geography and Statistics (IBGE) as an equivalent term for race. IBGE describes the color or race of the Brazilian population based on self-declaration.

**Table 2:** Characteristics, laboratory, screening, and confirmatory tests for HTLV-1/2 in HCV-infected individuals treated in Belém do Pará.

Patient	Age	Sex	AST (U/L)	ALT (U/L)	GGT (U/L)	anti-HCV	Viral Load (IU/mL)	HCV Genotype	HCV Subtype	ELISA anti-HTLV-1/2	INNO-LIA		*q* PCR		Result
											*gp46* *I*	*gp46* *II*	*Pol* *I*	*Pol* *II*	
1	35	M	54	59	120	+	850,000	1	1a	+	+	-	+	-	HTLV-1
2	66	M	50	39	204	+	1,208,800	1	1a	+	+	-	+	-	HTLV-1
3	50	F	176	170	47	+	850,000	1	NA	+	-	-	-	+	HTLV-2
4	72	F	26	25	17	+	364,974	NA	NA	+	+	-	-	-	HTLV-1

M: Male; F: Female; -: undetectable or nonreactive; +: detectable or reactive; AST: Aspartate amino transferase; ALT: Alanine amino transferase; GGT: Gamma glutamyl transferase; NA: not-available.

**Table 3:** Risk factors and clinical characteristics in individuals monoinfected with HCV and coinfected with HCV/HTLV-1/2 in Belém do Pará.

Risk Factors	Total	HCV	HCV/HTLV-1/2	*p*
	*n* = 192 (%)	*n* = 188 (%)	*n* = 4 (%)	
Tattoo				
No	143 (74.5)	140 (74.5)	3 (75.0)	1.000
Yes	49 (25.5)	48 (25.5)	1 (25.0)	
Blood transfusion				
No	143 (74.5)	141 (75.0)	2 (50.0)	0.269
Yes	49 (25.5)	47 (25.0)	2 (50.0)	
Year of transfusion	***n* = 49 (%)**	***n* = 47 (%)**	***n* = 2 (%)**	
Before 1993	27 (55.1)	25 (53.2)	2 (100.0)	
After 1993	17 (34.7)	17 (36.2)	0	- *
Not informed	5 (10.2)	5 (10.6)	0	
Illicit drugs				
No	144 (75.0)	141 (75.0)	3 (75.0)	
Yes	47 (25.0)	46 (24.5)	1 (25.0)	1.000
Not informed	1 (0.5)	1 (0.5)	0	
HCV RNA				
Detectable	72 (37.5)	68 (36.2)	4 (100,0%)	
Not detectable	31 (16.1)	31 (16.5)	0	0.313
Not informed	89 (46.4)	89 (47.3)	0	
Hepatitis				
Chronic hepatitis	131 (68.2)	128 (68.1)	3 (75.0)	
Not informed	61 (31.8)	60 (31.9)	1 (25.0)	- *
Cirrhosis				
No	119 (62.0)	115 (61.2)	4 (100.0)	
Yes	22 (11.5)	22 (11.7)	0	1.000
Not informed	51 (26.6)	51 (27.1)	0	

* Statistical analysis was not performed because the expected frequencies were low.

## Data Availability

Ethical restrictions may be applied to the public availability of the data. However, they can be obtained by contacting the corresponding authors.

## Abbreviations

The following abbreviations are used in this manuscript:

HTLV-1/2	Human T-lymphotropic virus-1/2
HCV	Hepatitis C virus
HIV	Human immunodeficiency virus
HBV	Hepatitis B virus
ALT	Alanine amino transferase
AST	Aspartate amino transferase
GGT	Gamma-Glutamyl Transferase
qPCR	quantitative polymerase chain reaction or real-time PCR
DNA	Deoxyribonucleic acid
RNA	Ribonucleic Acid
SUS	Unified Health System
